# The Effect of Molten Salt Infiltration on 2D SiC_f_/SiC Composite by Chemical Vapour Infiltration

**DOI:** 10.3390/ma15207182

**Published:** 2022-10-14

**Authors:** Yantao Gao, Hui Tang, Ke Li, Hao Yan

**Affiliations:** 1 School of Textiles and Fashion, Shanghai University of Engineering Science, Shanghai 201620, China; 2 School of Mechanical and Energy Engineering, Shanghai Technical Institute of Electronics & Information, Shanghai 201411, China; 3 Shanghai Institute of Applied Physics, Chinese Academy of Sciences, Shanghai 201800, China

**Keywords:** SiC_f_/SiC composites, molten salt infiltration, CT, microstructure

## Abstract

The SiC_f_/SiC composite manufactured by chemical vapour infiltration (CVI) is a kind of porous material. Liquid molten salt in a Molten Salt Reactor (MSR) may enter into the porous composites and affect their performance. Through the study of the internal pores in the material, the permeability behaviour of the material can be investigated, which is of great significance to the analysis of the properties of the material itself. However, there is less investigation on effects of molten salt infiltration on the internal pore structure of SiC_f_/SiC composites. In this paper, a molten salt infiltration experiment of 2D woven SiC_f_/SiC composites was implemented at 650 °C, 3 atm. SEM, CT and XRD were used to characterize it. The results indicated that the microstructure could be affected by partial molten salt infiltration and temperature change. The distribution of porosity of the composite showed an obvious transformation. The lattice spacing of SiC showed an increased tendancy of stress relaxation.

## 1. Introduction

The continuous SiC fibre reinforced SiC ceramic matrix composites, namely SiC_f_/SiC composites, are widely used in aviation, aerospace, energy, transportation, and other fields due to their excellent high-temperature resistance, oxidation resistance, and high specific strength [1,2,3].

In MSR, core structure materials are always used under extreme conditions such as high-temperature irradiation, molten salt corrosion and so on [4,5,6]. The properties of these materials under molten salt environment are critical in the development of new structural materials [7]. SiC_f_/SiC composite has the characteristics of low thermal expansion coefficient, low neutron absorption cross section, thermal shock resistance, corrosion resistance at high temperature, high strength, and is one of the candidate materials for reactor accident-resistant fuel [8]. Previous studies have shown that ceramic matrix composite is a complex porous structure. When it is exposed to high temperature molten salt above a certain pressure, the salt can enter into the pores in the composite material by liquid penetration or vapour phase diffusion. Thus, the pores inside the composite are filled [9]. Researchers have also found that after liquid salt enters the pores of porous materials, it will lead to salt crystallization under certain conditions and produce crystallization pressure inside the materials, leading to changes in the microstructure of the materials or direct destruction of the materials [10,11,12]. In the SiC_f_/SiC composites, there are pores of larger size among fibre bundles and pores of smaller size inside the fibre bundles [13]. Therefore, molten salt can also infiltrate into the internal pore structure of SiC_f_/SiC composites, affecting the microstructure of SiC_f_/SiC composites. It can be seen that molten salt can be immersed in the internal pores of the composite material, and the liquid salt impregnated in the porous structure material will interact with the composite material in a complex manner. The performance of the composite material will be affected when the composite receives the loading action, which will cause the internal structure of the composite material to change [14,15,16]. In this case, it is necessary to study the pore structure of SiC_f_/SiC composites before and after molten salt infiltration. In this test, 2LiF-BeF_2_ (FLiBe) salt was used for infiltration, and the samples were soaked for 20 h at 650 °C under 3 atm pressure. The SiC_f_/SiC composites used in the experiment were prepared by chemical vapour infiltration. A large number of pores exist in the composite material, including small pores inside the fibre bundle and large pores among the fibre bundles. For the study of pores, due to their non-uniformity, it is difficult for conventional imaging techniques to observe their 3D microstructure, and leads to wear or damage to the sample during the processing, so the available information is limited.

X-ray-computed tomography is a powerful nondestructive analysis tool, especially for some porous structures of composite materials [17]. Synchronous X-ray radiation CT has many advantages of high resolution, cross-sectional imaging and volume imaging [18]. Some scholars have successfully applied the synchronous x-ray radiation CT technology in the research on the SiC_f_/SiC composite and the failure process of SiC_f_/SiC composite materials [19,20,21,22]. In this study, synchronous radiation X-ray-computed tomography (SR-CT) was used to study the microstructure changes of SiC_f_/SiC composite before and after molten salt infiltration. XRD was performed to study the effect of molten salt infiltration on strain status. Finally, the micromechanical property of the matrix in the SiC_f_/SiC composite was measured by Nano indenter. It is helpful for us to understand the influence of molten salt infiltration on the microstructure and mechanical property of SiC_f_/SiC composite materials.

## 2. Materials and Methods

### 2.1. Description of 2D Composite Material

**Figure 1:**
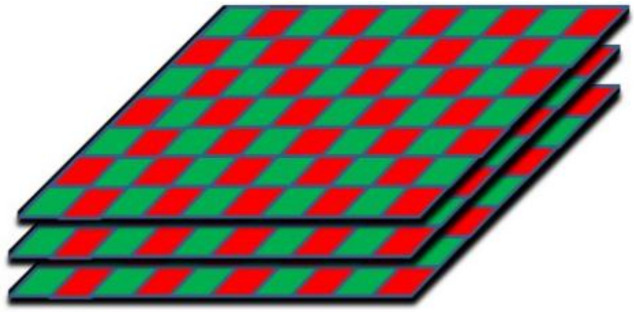
Schematic diagrams of stitched 2D woven preform.

2D-woven preform was made of domestic SiC fibre bundles produced by the Shanghai Institute of Applied physics, Chinese Academy of Sciences (Shanghai, China). It consists of two sets of fibre interwoven orthogonally in each layer and then several layers of weaves were built vertically upon each other (Figure 1). 

The SiC_f_/SiC composite material was fabricated by isothermal CVI at the Shanghai Institute of Ceramics, Chinese Academy of Sciences (Shanghai, China). Prior to CVI, a multilayered interphase was deposited in a similar way onto the surface of the preform. The multilayered interphase consists of alternating layers of 250 nm thick pyrolytic carbon (PyC), 100 nm thick SiC and 50 nm thick PyC. The fibre volume fraction of the composite was about 43% and residual porosity about 13%. Then the material was machined to small samples to facilitate high resolution CT characterization. 

### 2.2. Experimental Methods

#### 2.2.1. Molten Salt Infiltration Experiment

**Figure 2:**
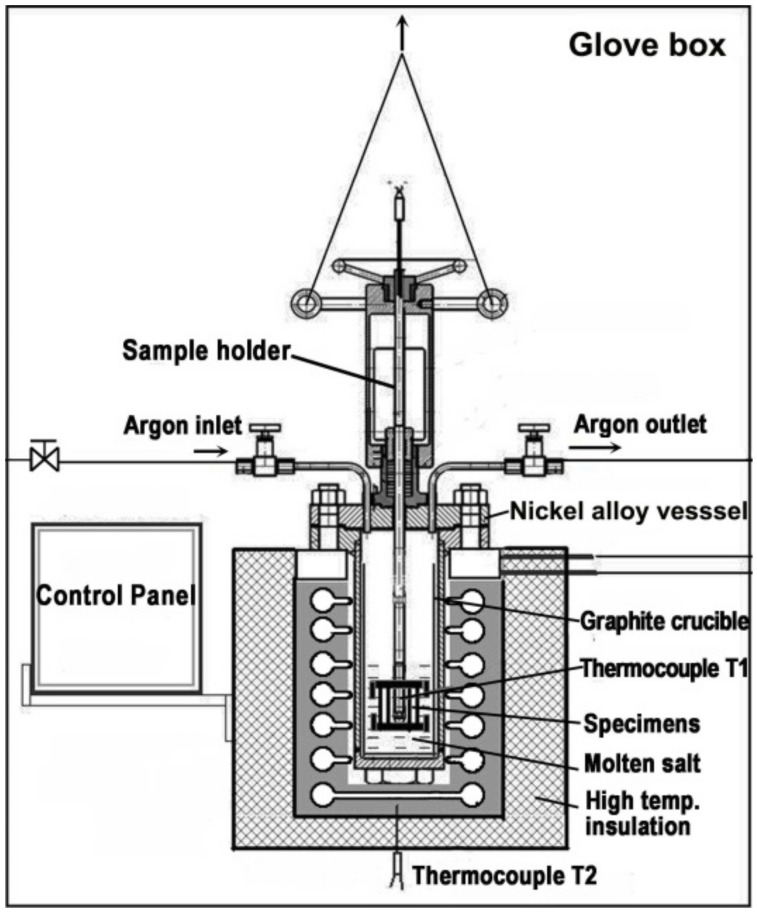
Schematic of the equipment used for molten salt infiltration experiment.

A total of 3 specimens of 3 mm × 3 mm SiC_f_/SiC composite were used in the experiment. A mixture of LiF and BeF_2_ salts (LiF-BeF_2_: 66–34 mol%, FLiBe (Manufactured by Shanghai Institute of Organic Chemistry, Chinese Academy of Sciences, Shanghai, China)) was used to prepare a eutectic salt. The experiment was conducted in a pressurized vessel, which was described in Reference [23], as shown in Figure 2. A sample holder which was used to put samples on was fixed to the lifting appliance. Argon gas served as a protective gas as well as a pressurized gas in the vessel. After heating the vessel to 650 °C to melt the salt, the specimens were immersed into molten FLiBe salt by descending the lifting appliance. After the experiment, the specimens were separated from the molten salt by raising the lifting appliance.

#### 2.2.2. SEM and X-ray Computed Tomography

The microstructures of the SiC_f_/SiC composite were examined by field-emission scanning electron microscope (FESEM, LEO 1530 VP, Zeiss, Germany). 

3D imaging was carried out at the X-ray imaging and biomedical application beam line station (BL13W1) beamline of Shanghai Synchrotron Radiation Facility (Shanghai, China). The experiment conditions (beam energy: 22 KeV, resolution: 3.25 μm) were set up. During the CT scan in one cycle, a total of 900 projection images were collected in each experiment. Section images were 3D reconstructed using the software ‘PITRE3’ developed by a researcher in BL13W1. Then the processed images were stacked into a 3D model by the commercial software ‘AVIZO’ 9.1. Similarly, the software was used to analyze the pores and calculate the porosity by threshold segmentation, grayscale processing and morphological algorithms of the model. The 3D pore model of the pores in the fibre bundles and the pores among the fibre bundles can be displayed and quantitative analysis can also be implemented.

#### 2.2.3. XRD Characterization

The crystal phases of the SiC_f_/SiC before and after molten salt infiltration were measured by X-ray diffraction (D8 Advance, Bruker, Germany) with a Cu Kα1 radiation source (λ = 1.5406 Å). In the experiment, reflected X-ray intensity was collected by a Lynx Eye XE counter (Bruker, Germany), using a continuous θ–2θ scans mode at a tube power of 40 kV/40 mA. The sample scan range was 20–90° (2θ) with a step size of 0.02° (2θ) at a rate of 0.15 s per step.

## 3. Results and Discussions

### 3.1. Morphology Characterization of SiC_f_/SiC Composite by SEM

**Figure 3:**
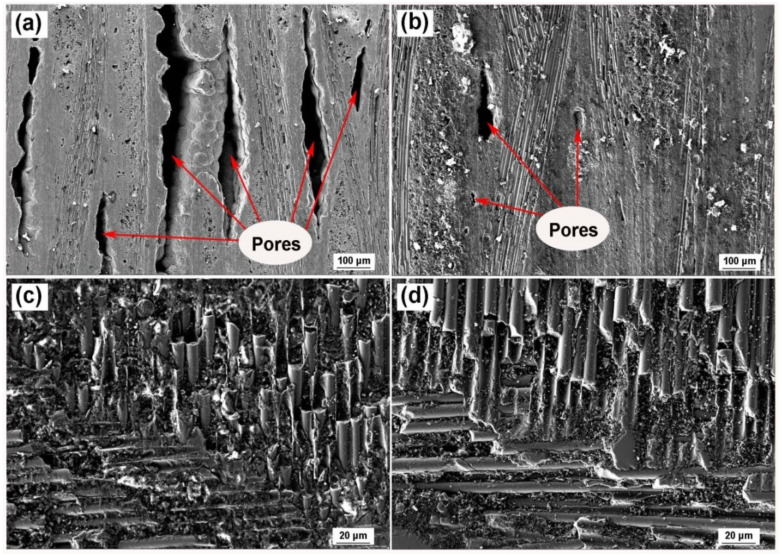
SEM morphology of SiC_f_/SiC composite. Cross-sectional images of (**a**) as-received sample, and (**b**) salt-infiltrated sample. Surface images of (**c**) as-received sample, and (**d**) salt-infiltrated sample.

Figure 3 showed surface topography of SiC_f_/SiC composite before and after molten salt treatment by SEM. In Figure 3a, for as-received sample, pores presented in a long strip shape with a length between several hundred micrometers to several centimeters and a width from dozens to several hundred micrometers. After the molten salt infiltration test, these pores were filled with salt and became shorter and narrower, or even disappeared, as described in Figure 3b. For surface images in Figure 3c,d, there were no obvious changes as there were no large pores, while some fragments on the surface were washed away after the molten salt infiltration test. 

### 3.2. Microstructure SR-CT Characterization of SiC_f_/SiC Composite without and with Molten Salt Treatment

**Figure 4:**
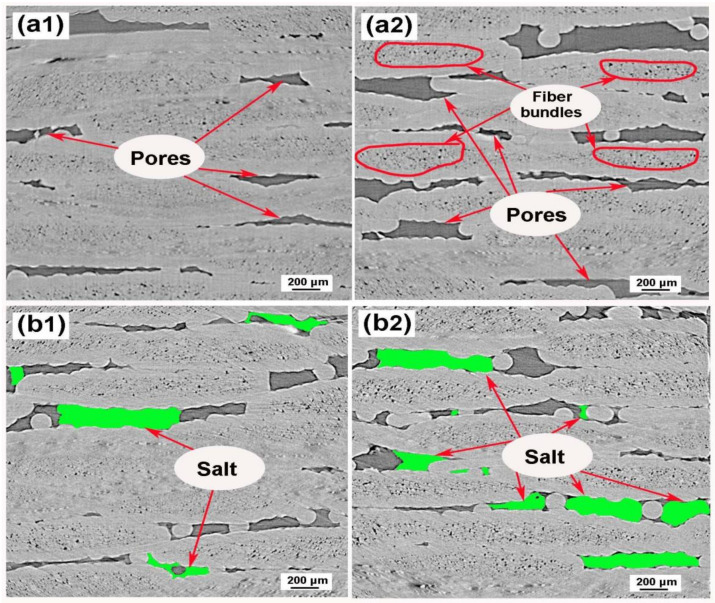
Cross-sectional µ-CT slices of SiC_f_/SiC composite (**a1**,**a2**) without molten salt infiltration, (**b1**,**b2**) with molten salt infiltration.

Figure 4(a1,a2) were two slices which represented the cross-sections in the SiC_f_/SiC composite before molten salt treatment. From them, the pore structures can be observed clearly. In the process of molten salt treatment, the infiltrated molten salt crystallized in the pores when it cooled down. The salt distribution in the SiC_f_/SiC composite was shown in Figure 4(b1,b2). It can be seen that it was unordered or inhomogeneous in the pore structures. And no salt appeared in some pores. The reason should be attributed to the complex structure of pores in SiC_f_/SiC composite. The Washburn’s formation (Equation (1)) related the pressure difference (ΔP) to the corresponding pore size (D) using the surface tension of infiltration liquid (σ) and the contact angle (θ) between the solid and infiltration liquid:

(1) $$\Delta P=\frac{-4\sigma \cos\theta }{D}$$

Therefore, molten salt can only penetrate some pores under controlled pressure. And the infiltrated molten salt will shrink when it crystallizes. Additionally, the closed pores in SiC_f_/SiC composite cannot be infiltrated. Finally, the salt distribution in the 2D SiC_f_/SiC composite assumed irregularity as shown in Figure 4(b1,b2). 

### 3.3. X-ray Diffraction Analysis

**Figure 5:**
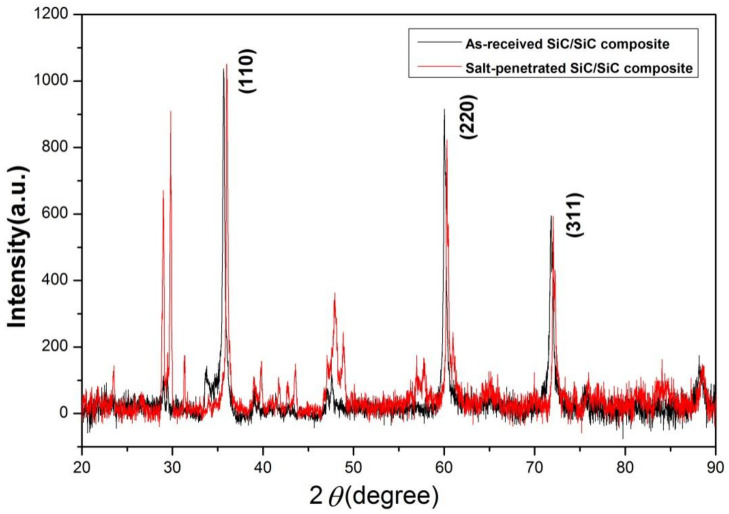
XRD patterns of SiCf/SiC composite before and after molten salt treatment.

Figure 5 showed XRD patterns of the as-received and salt-penetrated SiC_f_/SiC composites. It was clear that the peak of SiC in the salt-penetrated SiC_f_/SiC composite sample presented a significant shift to the higher diffraction angle compared to that of the same sample without molten salt infiltration, demonstrating the appearance of a smaller strain in the materials. Usually, molten salt infiltration will raise strain in the porous materials as the squeeze occurs in the crystallization. However, internal stress could exist in the manufacturing process. In the molten salt treatment process, the internal stress releases as the molten salt and pore structure interact under high temperature. Therefore, the strain displayed a decrease trend. 

**Figure 6:**
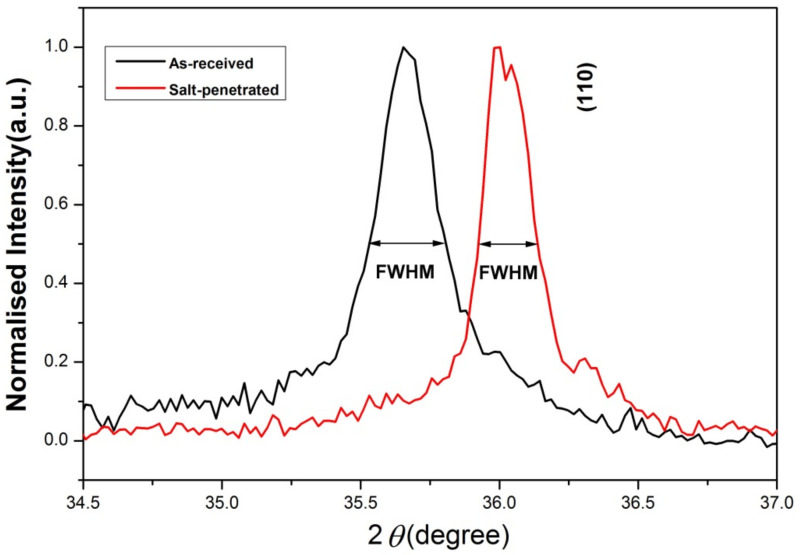
Comparison of (110) diffraction peaks of SiC_f_/SiC composite before and after molten salt infiltration.

The full width at half-maximum (FWHM) values of the (110) diffraction peak of SiC_f_/SiC composite were calculated to understand the change of crystallite size after molten salt infiltration. As shown in Figure 6 and Table 1, the FWHM of the as-received sample was 0.2757°, while for salt-penetrated sample was 0.2131°. A decrease in FWHM of (110) diffraction peak indicated an increasing structural order of SiC_f_/SiC composite after molten salt infiltration. The crystallite size of SiC_f_/SiC composite is mainly related to its FWHM value of diffraction peak, which can be calculated by a Scherrer Equation, which is given by [24].

(2) $$L_{hkl}=\frac{K\lambda }{\beta \cos\theta }$$

where *L_hkl_* is the crystallite size of (*hkl*) diffraction plane, *λ* is the wavelength of the incident X-ray, θ is the diffraction angle of (*hkl*) diffraction plane, *β* is the value of the FWHM, and *K* is a constant equal to 0.89 for SiC_f_/SiC composite material. 

According to Equation (2) and Table 1, the crystallite size of the (110) diffraction plane for the as-received sample of SiC_f_/SiC composite was 29.93 nm, while for salt-penetrated sample was 38.76 nm. The reason could be attributed to stress decreasing in the surface of material. This reason matched the right shift of wave peaks.

### 3.4. Comparative Analysis of Pore Structures in SiC_f_/SiC Composite before and after Molten Salt Infiltration 

**Figure 7:**
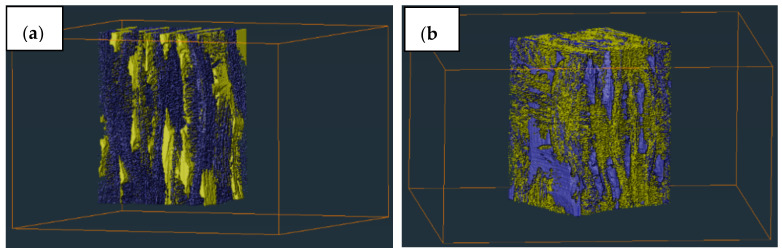
Pore structures comparison of SiC_f_/SiC composite (**a**) before and (**b**) after molten salt infiltration.

3D images of overall pores inside the composite material before and after molten salt treatment were restructured as shown in Figure 7. The result indicated that molten salt infiltration had some influence on the porosity of the SiC_f_/SiC composite. According to the results calculated in Avizo, the porosity of SiC_f_/SiC composite before molten salt infiltration was 10.66%, the volume of small pores accounted for 30.71%, and the volume of large pores accounted for 69.29%. However, the overall porosity and the proportion of small pores in the SiC_f_/SiC composite presented a slight rising trend after infiltration. The porosity of SiC_f_/SiC composite increased to 11.27% and the volume of small pores accounted for 36.28% and the volume of large pores accounted for 63.72%. Table 2 showed the proportion of pores in number divided according to pore size. It can be seen that the number of small pores in the fibre bundle increased after infiltration, and molten salt treatment had a significant effect on the small pores after infiltration. The results also illustrated that the porosity of SiC_f_/SiC composite increased after receiving molten salt infiltration, the proportion of small pores in the pores increased after molten salt infiltration, and the molten salt treatment had a relatively great influence on the small pores inside the fibre bundles.

**Figure 8:**
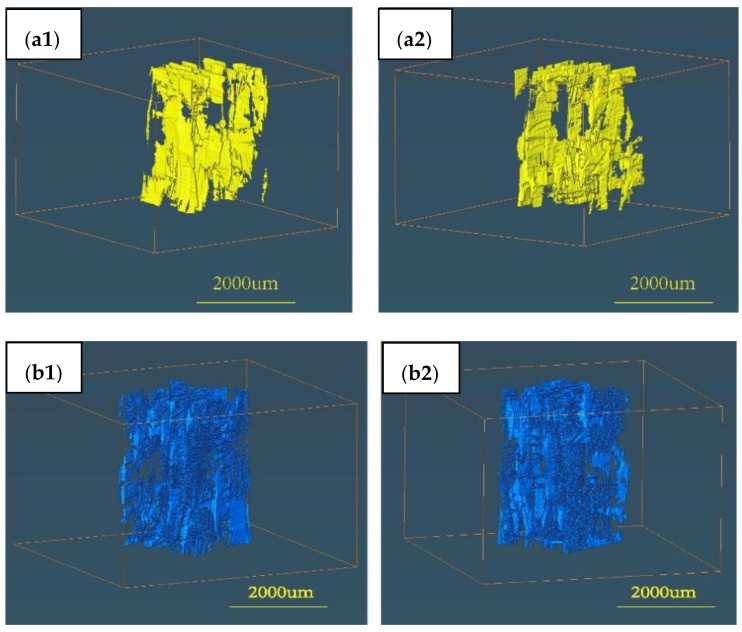
Large pores (≥10μm) comparison of SiC_f_/SiC composite (**a1**,**a2**) without molten salt infiltration and (**b1**,**b2**) with molten salt infiltration.

Figure 8(a1,a2) were the 3D images of the large pores before infiltration and Figure 8(b1,b2) were the 3D images of the large pores after infiltration. Figure 8(a1,a2) indicated that large pores existed between fibre bundles, especially between adjacent layers. It also can be seen that the number of large pores increased obviously after infiltration, but the results calculated by the software ‘AVIZO’ showed that the volume proportion of the large pores decreased after infiltration. The reason could be that some large pores become thinner as stress release impelled space reduction in pore structures. However, a few pores could increase as salt remained, which occurred on the surface.

**Figure 9:**
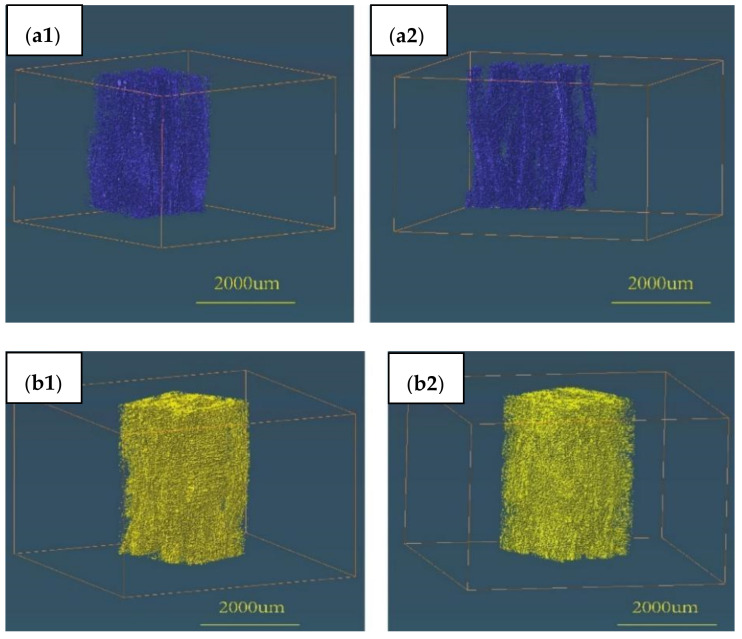
Small pores (<10 μm) comparison of SiC_f_/SiC composite (**a1**,**a2**) without molten salt infiltration and (**b1**,**b2**) with molten salt infiltration.

Comparing the small pores before and after molten salt treatment in Figure 9, it can be seen that the small pores significantly densified after molten salt infiltration. This should be also attributed to stress release as it was heated again in the infiltration. As prestress generated in the manufacturing process decreased, some small pores would expand. Therefore, new small pores appeared, which led to the increase of volume proportion of small pores in the SiC_f_/SiC composite. 

## 4. Conclusions

A molten salt infiltration experiment was carried out on 2D SiCf/SiC composite samples. It can be concluded that molten salt infiltration and temperature change could have significant effects on pore structures. The porosity of SiC_f_/SiC composites showed a slight rising trend after it was penetrated by molten salt. The pore distribution after molten salt infiltration was mainly affected by stress release and molten salt infiltration. Although the molten salt infiltration reduced the size of the pores in some pores as the squeeze between salt and SiC fibre or matrix, stress release would impel some pores to become bigger or new small pores to appear in the SiC_f_/SiC composite. An XRD pattern showed that the strain displayed a decrease trend and an increase in crystal size. The results agreed with each other. To be noted, molten salt infiltration was influenced by pore structure, which closely related to the preform of the SiC_f_/SiC composite. Therefore, it is important to take the preform of the composite into account when considering molten salt infiltration. 

## Figures and Tables

**Table 1:** Comparison of the value of 2θ of (110) diffraction peaks and Full Width Half-Maximum (FWHM) of SiC_f_/SiC composite before and after molten salt infiltration.

2θ (°) As Received	2θ (°) Salt-Penetrated	FWHM (°) As Received	FWHM (°) Salt-Penetrated
35.6540	36.0019	0.2757	0.2131

**Table 2:** The proportion of pores in SiC_f_/SiC composite.

	Less than 10 μm	More than 10 μm
Proportion of pores before infiltration	71.36%	28.64%
Proportion of pores after infiltration	80.17%	19.83%

## Data Availability

The datasets generated and/or analyzed during the current study are available from the corresponding author on reasonable request.

## Nomenclatures

SiC_f_/SiC composites	SiC fibre reinforced SiC matrix composites;
CT	computed tomography;
SR-CT	synchrotron radiation X-ray computed tomography;
XRD	X-ray diffraction;
SEM	scanning electron microscopy;
CVI	chemical vapour infiltration;
MSR	Molten Salt Reactor;
PyC	pyrolytic carbon
ΔP	the pressure difference inside and outside of pores;
D	the diameter of the infiltrated pore;
σ	the surface tension of infiltration liquid;
θ	the contact angle between infiltration liquid and solid.
FWHM	the full width at half maximum
